# Role of HNF4α-cMyc Interaction in CDE Diet–Induced Liver Injury and Regeneration

**DOI:** 10.1016/j.ajpath.2024.03.008

**Published:** 2024-07

**Authors:** Manasi Kotulkar, Julia Barbee, Diego Paine-Cabrera, Dakota Robarts, Maura O’Neil, Udayan Apte

**Affiliations:** Department of Pharmacology, Toxicology, and Therapeutics, University of Kansas Medical Center, Kansas City, Kansas

## Abstract

Hepatocyte nuclear factor 4 alpha (HNF4α) is a nuclear factor essential for liver function that regulates the expression of cMyc and plays an important role during liver regeneration. This study investigated the role of the HNF4α-cMyc interaction in regulating liver injury and regeneration using the choline-deficient and ethionine-supplemented (CDE) diet model. Wild-type (WT), hepatocyte-specific HNF4α-knockout (KO), cMyc-KO, and HNF4α-cMyc double KO (DKO) mice were fed a CDE diet for 1 week to induce subacute liver injury. To study regeneration, normal chow diet was fed for 1 week after CDE diet. WT mice exhibited significant liver injury and decreased HNF4α mRNA and protein expression after CDE diet. HNF4α deletion resulted in significantly higher injury with increased inflammation, fibrosis, proliferation, and hepatic progenitor cell activation compared with WT mice after CDE diet but indicated similar recovery. Deletion of cMyc lowered liver injury with activation of inflammatory genes compared with WT and HNF4α-KO mice after CDE diet. DKO mice had a phenotype comparable to that of the HNF4α-KO mice after CDE diet and a complete recovery. DKO mice exhibited a significant increase in hepatic progenitor cell markers both after injury and recovery phase. Taken together, these data show that HNF4α protects against inflammatory and fibrotic changes after CDE diet–induced injury, which is driven by cMyc.

## Graphical abstract

**Figure:**
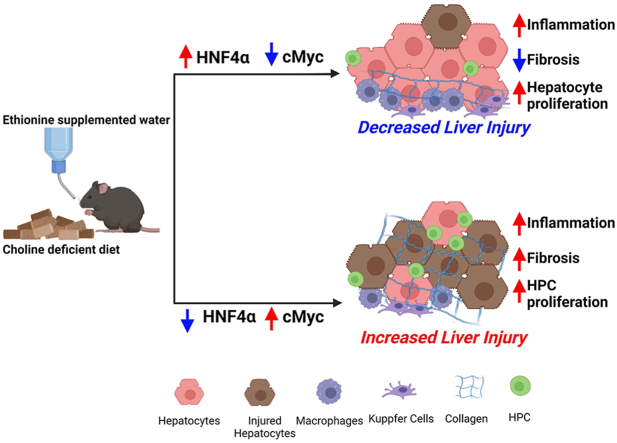

Hepatocyte nuclear factor 4 alpha (HNF4α) is a key nuclear receptor expressed by hepatocytes. HNF4α is important for liver development and the maintenance of mature liver function, and it plays an important role in maintaining hepatocyte differentiation.^1^ Decreased HNF4α results in the loss of hepatic function and a de-differentiated phenotype.^2^ Ablation of HNF4α in the liver induces spontaneous proliferation of hepatocytes via induction of mRNA and protein expression of several pro-mitogenic genes, including cMyc.^3^ A recent study investigated the role of HNF4α-cMyc interaction in liver regeneration after drug-induced acute liver injury.^4^ HNF4α interacts with Nrf2 and contributes to recovery from acetaminophen-induced liver injury by down-regulating the expression of cMyc.

The choline-deficient and ethionine-supplemented (CDE) diet is a model used to induce chronic liver injury in mice. Dietary deficiency of choline results in disrupted fat metabolism and the secretion of very-low-density lipoproteins, contributing to steatosis. Ethionine is a hepatocarcinogen that, in combination with the choline-deficient diet, leads to hepatic fat loading, inflammation, fibrosis, hepatic progenitor cell (HPC) response, and, eventually, development of hepatocellular carcinoma.^5^ Proliferation is a key event in liver regeneration after injury. In a standard regeneration process, hepatocytes and cholangiocytes replenish their respective cell types and restore the hepatic mass. However, during chronic liver injury, when hepatocyte proliferation is inhibited or delayed, HPC aids in liver regeneration by first proliferating and later differentiating into functional hepatocytes.^6^ After a CDE diet–induced injury, inflammation and activation of HPC are observed, making it a physiologically relevant model of chronic liver injury.

The current study investigated the role of HNF4α-cMyc interaction in CDE diet–induced liver injury. Deletion of HNF4α increased subacute liver injury induced by CDE diet, and deletion of cMyc protected against the injury.

## Materials and Methods

### Animals, Treatment, and Tissue Harvesting

All animals were housed in facilities accredited by the Association for Assessment and Accreditation of Laboratory Animal Care at the University of Kansas Medical Center under a standard 12 hours’ light/dark cycle with free access to chow and water. All studies were approved by the Institutional Animal Care and Use Committee at the University of Kansas Medical Center; the committee abides by the ARRIVE (Animal Research: Reporting of In Vivo Experiments) statement. Two-month–old male C57BL/6J mice were purchased from The Jackson Laboratories (Bar Harbor, ME) and used for the initial analysis. HNF4α-floxed, cMyc-floxed, and HNF4α-cMyc double floxed mice were injected intraperitoneally with AAV8-TBG-CRE to generate hepatocyte-specific HNF4α-knockout (KO), cMyc-KO, and double KO mice, respectively, as described previously.^3^ Floxed mice injected intraperitonially with AAV8-TBG-GFP were used as controls. To study liver injury, these mice were fed a choline-deficient diet (TD.04523; Envigo, Indianapolis, IN) supplemented with 0.15% ethionine in drinking water (146170100; Acros Organics, Thermo Scientific Chemicals, Geel, Belgium) for 1 week. To study regeneration and recovery, mice were switched back to a normal chow diet and drinking water for 1 week after 1 week of a CDE diet. After euthanasia, liver and blood samples were collected and processed for further analysis, as described previously.^3^

### Protein Isolation and Western Blot Analysis

Western blot analysis was performed by using whole liver lysates for HNF4α (PP-H1415-00; Perseus Proteomics, Tokyo, Japan) and cMyc (56055; Cell Signaling Technology, Danvers, MA), as described previously.^7^ GAPDH (2118; Cell Signaling Technology) was used as a housekeeping control. The Odyssey Fc system was used to image the Western blots (LI-COR, Lincoln, NE).

### Real-Time PCR

RNA isolation and its cDNA conversion were conducted as previously described.^7^ Real-time quantitative PCR (qPCR) analysis was done on a CFX384 system (Bio-Rad, Hercules, CA). Then, 100 ng of cDNA was used per reaction to measure the mRNA expression of genes of interest using the manufacturer’s protocol (Thermo Fisher Scientific, Waltham, MA). The 18s gene expression in the same samples was used to normalize the cycle threshold values, as described previously.^8^ The cycle threshold values for the 1-week CDE diet and recovery groups were compared with those of the normal diet given the control group of the respective phenotype. The primers used for real-time PCR are listed in Table 1.

**Table 1:** Primers Used in This Study

Gene	Forward primer	Reverse primer
*Hnf4a*	5′-CAGTGTCGTTACTGCAGGCTT-3′	5′-GCTGTCCTCGATGCTTGACC-3′
*Ccnd1*	5′-GAATTCTATGACCCCTTGACCCC-3′	5′-TGGTGTTGGGTAAGAGGTTG-3′
*Myc*	5′-GGTGTTTGAAGGCTGGATTTC-3′	5′-GATGAAATAGGGCTGTACGGAG-3′
*Dio1*	5′-GTTGCACCTGACCTTCATTTC-3′	5′-CCACGTTGTTCTTAGAAGCCC-3′
*F12*	5′-GCCATTTTCCCTTTCAGTACC-3′	5′-TCTTTCACTTTCTTGGGCTCC-3′
*Ect2*	5′-TCACTCTTGCTTCAACCTGC-3′	5′-CGTGTTACCATCTCCTTCTGAG-3′
*Adgre1*	5′-CTGCACCTGTAAACGAGGCTT-3′	5′-TTGAAAGTTGGTTTGTCCATTGC-3′
*Il1b*	5′-ACGGACCCCAAAAGATGAAG-3′	5′-TTCTCCACAGCCACAATGAG-3′
*Ifng*	5′-ATGAAGGCTACACACTGGATC-3′	5′-CCATCCTTTTGCCAGTTCCTC-3′
*Tnfa*	5′-CCCTCACACTCAGATCATCTTCT-3′	5′-GCTACGACGTGGGCTACAG-3′
*Il10*	5′-GCTCTTACTGACTGGCATGAG-3′	5′-CGCAGCTCTAGGAGCATGTG-3′
*Cd163*	5′-TGGGTGGGGAAAGCATAACT-3′	5′-AAGTTGTCGTCACACACCGT-3′
*Clec4f*	5′-TTGGAGACCTGAGTGGAATAAAG-3′	5′-TAGTCCCTAAGCCTCTGGATAG-3′
*Ccr2*	5′-ATGCAAGTTCAGCTGCCTGC-3′	5′-ATGCCGTGGATGAACTGAGG-3′
*Vsig4*	5′-CCCTGGCTTCCTTTCTTCTTA-3′	5′-GCTGTCAGGCATGATAAA-3′
*Des*	5′-ATGCAGCCACTCTAGCTCGTA-3′	5′-CTCGTTGAGGGATTCGATTCTG-3′
*Tgfb1*	5′-AGCTGGTGAAACGGAAGCG-3′	5′-GCGAGCCTTAGTTTGGACAGG-3′
*Col1a1*	5′-ATGTTCAGCTTTGTGGACCTC-3′	5′-CAGAAAGCACAGCACTCGC-3′
*Col1a2*	5′-GGTGAGCCTGGTCAAACGG-3′	5′-ACTGTGTCCTTTCACGCCTTT-3′
*Col3a1*	5′-CCTGGCTCAAATGGCTCAC-3′	5′-CAGGACTGCCGTTATTCCCG-3′
*Ccnb1*	5′-TTGCAGTGAGTCACGTAGAC-3′	5′-CCTCCAGTTGTCGGAGATAAG-3′
*Ccnd1*	5′-GCCCTCCGTATCTTACTTCAAG-3′	5′-GCGGTCCAGGTAGTTCATG-3′
*Ccne1*	5′-GTGGCTCCGACCTTTCAGTC-3′	5′-CACAGTCTTGTCAATCTTGGCA-3′
*Krt19*	5′-GTTCTCAGACCTGCGTCC-3′	5′-TGACAAAATGCGTACTGAAC-3′
*Epcam*	5′-GCGGCTCAGAGAGACTGTG-3′	5′-CCAAGCATTTAGACGCCAGTTTT-3′
*Sox9*	5′-AGTACCCGCATCTGCACAAC-3′	5′-AGGAAGGGTCTCTTCTCGCT-3′
*18s*	5′-ACGGAAGGGCACCACCAGGA-3′	5′-TTTAGTATTGGACGCTGCCC-3′

### Staining Procedures

Paraffin-embedded 5 μmol/L liver tissue sections were used for hematoxylin and eosin and immunohistochemical staining of HNF4α (PP-H1415-00, 1:500; Perseus Proteomics), F4/80 (70076, 1:200; Cell Signaling Technology), α-smooth muscle actin (19245, 1:500; Cell Signaling Technology), Ki-67 (12202, 1:400; Cell Signaling Technology), and EpCAM (71916; Abcam, Cambridge, United Kingdom) as previously described.^7^^,^^9^ Formalin-fixed, paraffin-embedded tissue slides were stained with Picrosirius red as previously described.^10^ ImageJ software version 1.54i (NIH, Bethesda, MD; *http://imagej.nih.gov/ij*) was used to quantify the integrated density of positive staining above a constant threshold.

### Serum Alanine Aminotransferase Levels and Injury Scoring

Serum alanine aminotransferase (ALT) levels were measured by using a Pointe Scientific ALT Assay kit by Thermo Fisher Scientific according to the manufacturer’s protocol. Hematoxylin and eosin–stained liver tissues were scored for injury using the Batts-Ludwig grading of disease activity by a board-certified pathologist at the University of Kansas Medical Center.

### Statistical Analysis

Data presented in the form of bar graphs show the means ± SEM. GraphPad Prism 9 (GraphPad Software, La Jolla, CA) was used to graph and calculate statistics. Two-way analysis of variance and a *t*-test were applied to all analyses, with *P* < 0.05 considered significant. For all the experiments, three to five mice were used per group.

## Results

### Decreased HNF4α Expression after 1 Week of CDE Diet

**Figure 1:**
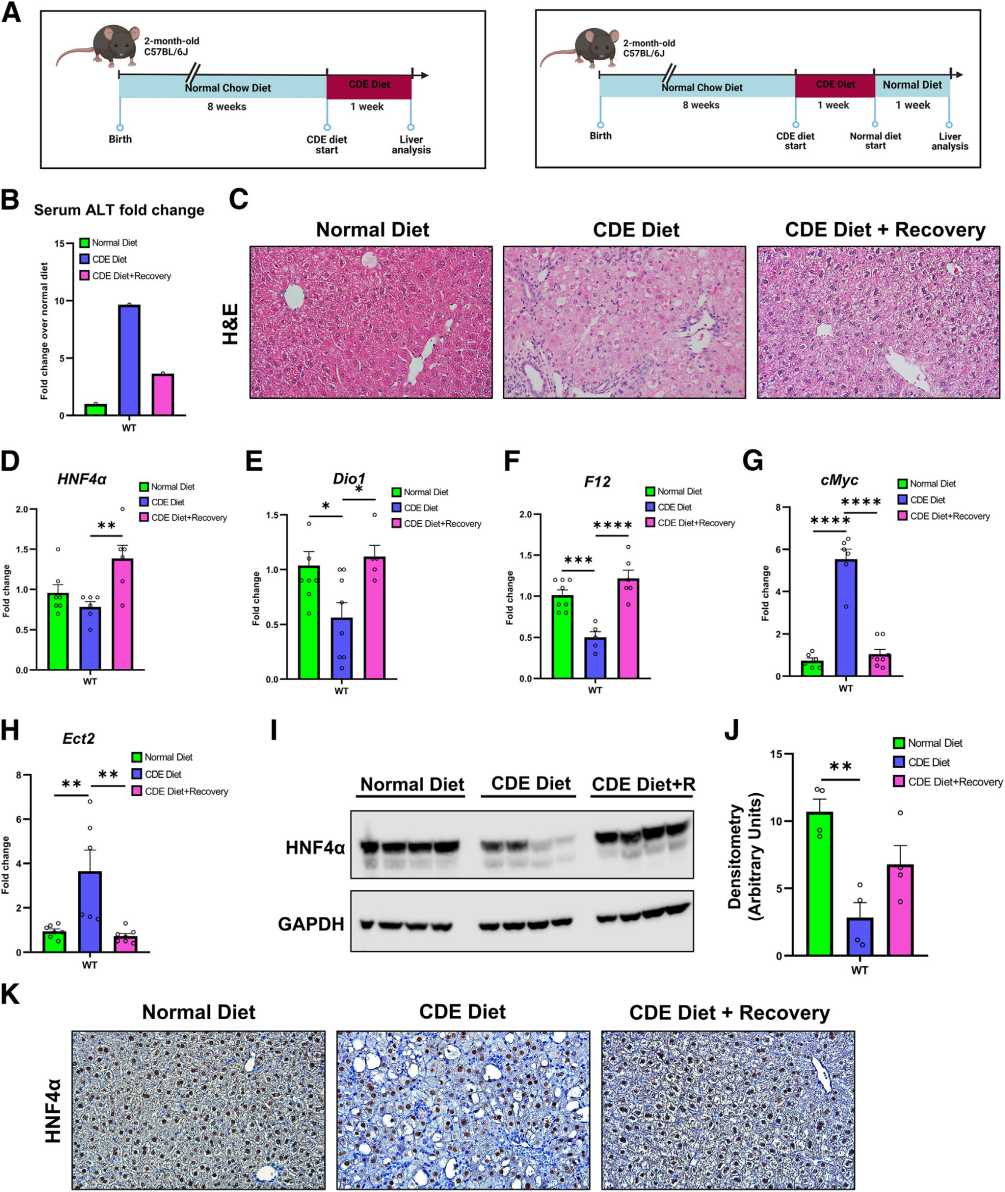
Decreased hepatocyte nuclear factor 4 alpha (HNF4α) expression after 1 week of a choline-deficient and ethionine-supplemented (CDE) diet. Study design (**A**), serum alanine aminotransferase (ALT) fold change (**B**), and representative photomicrographs of hematoxylin and eosin (H&E) staining (**C**). Real-time PCR analysis of *Hnf4a* (alias *HNF4α*) (**D**), *Dio1* (**E**), *F12* (**F**), *Myc* (alias *cMyc*) (**G**), and *Ect2* (**H**). Western blot analysis (**I**), densitometric analysis for HNF4α (**J**), and representative photomicrographs (**K**) of HNF4α immunohistochemistry of C57BL/6J mice after CDE diet–induced injury and regeneration. Bars represent means ± SEM. *n* = 3 to 5. ∗*P* < 0.05, ∗∗*P* < 0.01, ∗∗∗*P* < 0.001, ∗∗∗∗*P* < 0.0001. Original magnification: ×400 (**C** and **K**). Diet+R, diet plus recovery.

Two-month–old male C57BL/6J mice were fed a CDE diet for 1 week to study the injury response. To determine the regeneration response upon injury, these mice were allowed to recover on a normal diet for 1 week after the CDE diet (Figure 1A). The CDE diet resulted in an almost 10-fold increase in serum ALT levels, which were reduced after 1 week of recovery (Figure 1B). Serum ALT data were corroborated by hematoxylin and eosin staining, which showed disrupted liver histology with moderate fat accumulation and the presence of ductular reactions after CDE diet. During the recovery period, mice exhibited restored histology similar to that of the normal diet control mice (Figure 1C). *HNF4α* mRNA remained unchanged. However, genes positively regulated by HNF4α, including *Dio1* and *F12*, were significantly down-regulated, whereas genes negatively regulated by HNF4α, including *cMyc* and *Ect2*, were significantly up-regulated after CDE diet. During recovery on a normal diet, *HNF4α, Dio1*, and *F12* mRNA expression returned to control levels, and *cMyc* and *Ect2* mRNA expression was reduced to those of the normal diet control mice (Figure 1, D–H). Consistent with these results, Western blot analysis showed decreased protein expression of HNF4α after the CDE diet (Figure 1, I and J). The Western blot results were validated by HNF4α immunohistochemistry (Figure 1K).

### Significantly Higher Liver Injury after 1 Week of the CDE Diet

**Figure 2:**
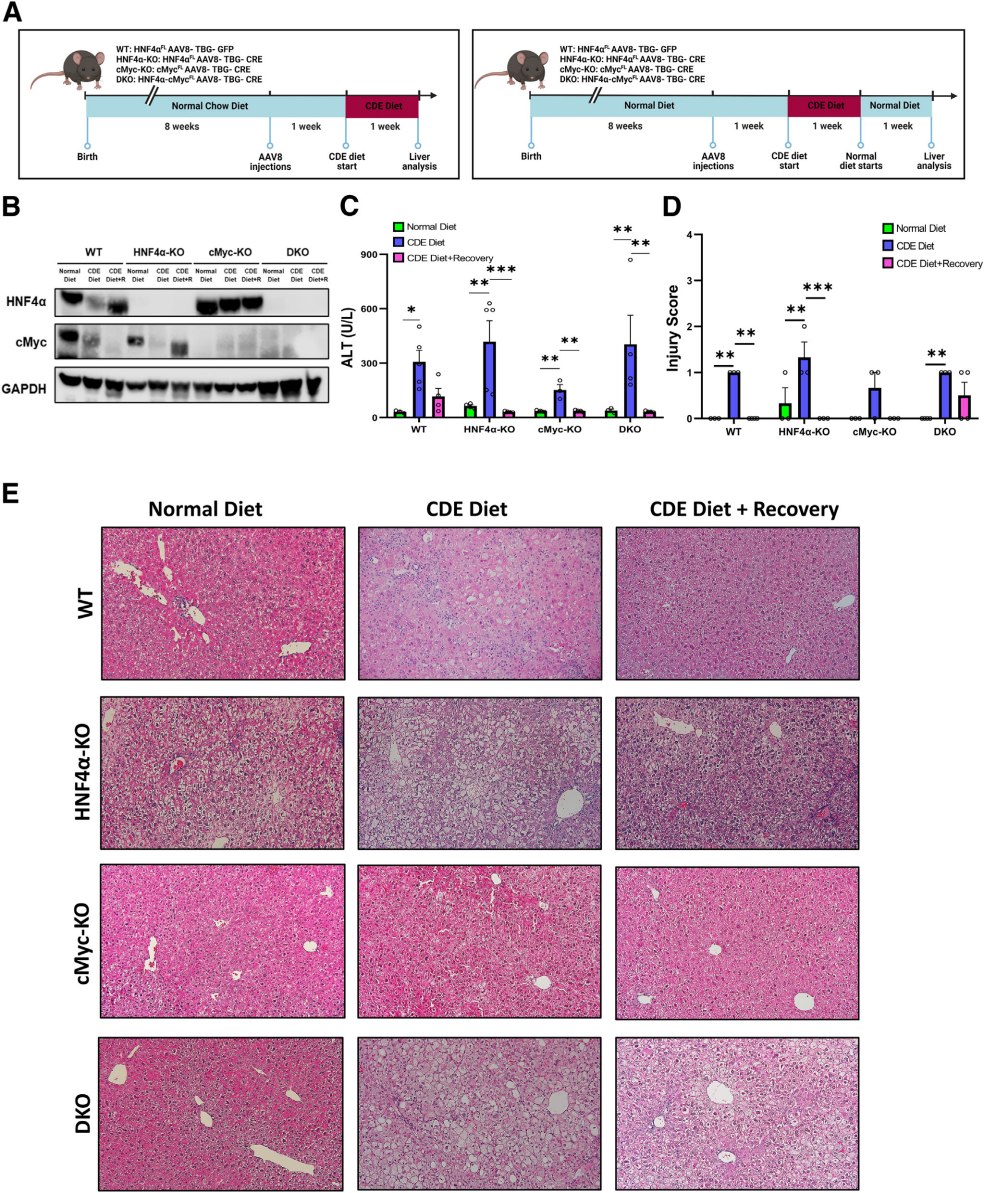
Significantly higher liver injury after 1 week of a choline-deficient and ethionine-supplemented (CDE) diet. Study design (**A**), confirmatory Western blot analysis (**B**), serum alanine aminotransferase (ALT) levels (**C**), injury score (**D**), and representative photomicrographs of hematoxylin and eosin staining (**E**) in wild-type (WT), hepatocyte nuclear factor 4 alpha (HNF4α)-knockout (KO), cMyc-KO, and double KO (DKO) mice after CDE diet–induced injury and regeneration. Bars represent means ± SEM. *n* = 3 to 5. ∗*P* < 0.05, ∗∗*P* < 0.01, ∗∗∗*P* < 0.001. Original magnification: ×200.

Liver injury and recovery were studied in wild-type (WT), HNF4α-KO, cMyc-KO, and double KO (DKO) mice after 1 week of CDE diet followed by 1 week of recovery on a normal diet (Figure 2, A and B). All four genotypes (WT, HNF4α-KO, cMyc-KO, and DKO mice) showed significantly high serum ALT levels, indicative of increased liver injury, after 1 week of a CDE diet. HNF4α-KO mice showed more injury, whereas the cMyc-KO mice experienced less injury than the WT mice. Injury was significantly resolved in all mice after 1 week of recovery on a normal diet (Figure 2C). Hematoxylin and eosin–stained liver tissues were examined and scored for injury by a board-certified pathologist (M.O.) (Figure 2D). Injury score data corroborated with the ALT data. HNF4α-KO mice had higher injury scores and cMyc-KO mice had overall lower injury scores after 1 week of the CDE diet compared with that in the WT control mice. Significant changes in histology, including fat accumulation observed in the staining images, were consistent with the injury data. Increased hepatocyte mitoses were observed in HNF4α-KO mice after 1 week of CDE diet. DKO mice exhibited centrilobular ballooning and cholestatic effects after feeding with the CDE diet. The histologic features were restored during the recovery phase (Figure 2E).

### Increased Inflammation in cMyc-KO Mice after 1 Week of the CDE Diet

**Figure 3:**
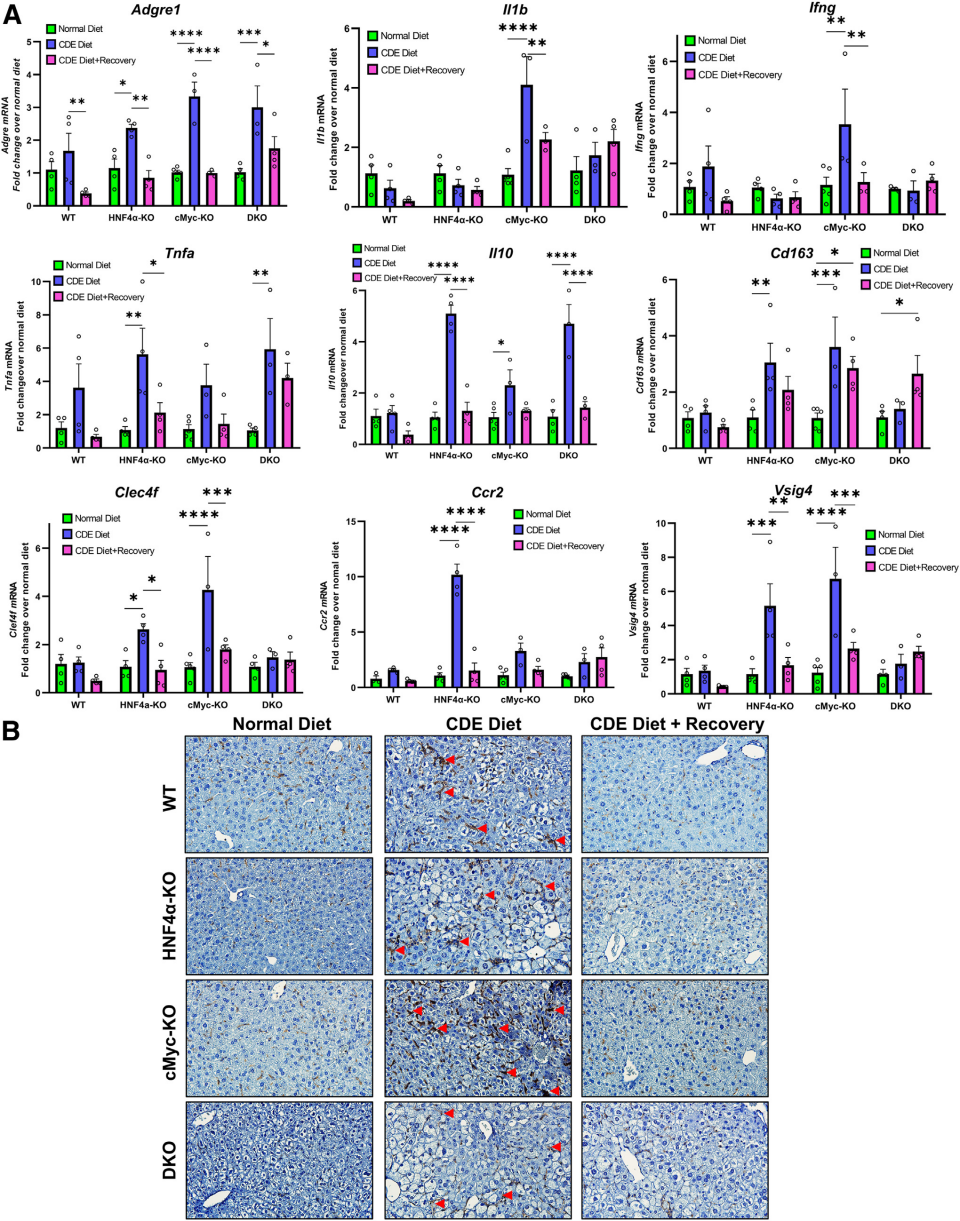
Increased inflammation in cMyc-knockout (KO) mice after 1 week of a choline-deficient and ethionine-supplemented (CDE) diet. Real-time PCR analysis of *Adgre*, *Il1b*, *Ifng*, *Tnfa, Il10, Cd163, Clef4f, Ccr2,* and *Vsig4* (**A**) and representative photomicrographs of F4/80 immunohistochemistry in wild-type (WT), hepatocyte nuclear factor 4 alpha (HNF4α)-KO, cMyc-KO, and double KO (DKO) mice (**B**) after CDE diet–induced injury and regeneration. **Red arrowheads** indicate F4/80-positive cells. Bars represent means ± SEM. *n* = 3 to 5. ∗*P* < 0.05, ∗∗*P* < 0.01, ∗∗∗*P* < 0.001, ∗∗∗∗*P* < 0.0001. Original magnification: ×400.

The status of inflammation was examined using qPCR analysis for several inflammation markers after 1 week of CDE diet and 1 week of recovery. HNF4α-KO mice showed significantly higher expression of inflammation mediator genes, including *Il1b, Ifng, Tnfa,* and *Il10*, and gene markers of cells involved in inflammation response, including *Adgre1,Cd163, Clef4, Ccr2,* and *Vsig4,* during the injury phase. Expression of inflammatory genes was significantly higher in cMyc-KO mice compared with WT and HNF4α-KO mice. In DKO mice, the induction of inflammatory genes was comparable to that of HNF4α-KO mice (Figure 3A). The mRNA data were further supported by F4/80 immunohistochemistry staining. Among the four genotypes, cMyc-KO mice showed significantly higher F4/80–positive cells after being fed the CDE diet (Figure 3B). The increased mRNA and protein expression of inflammation markers during CDE diet–induced injury was significantly decreased during the recovery phase in all groups.

### Increased Fibrosis in HNF4α-KO Mice after 1 Week of the CDE Diet

**Figure 4:**
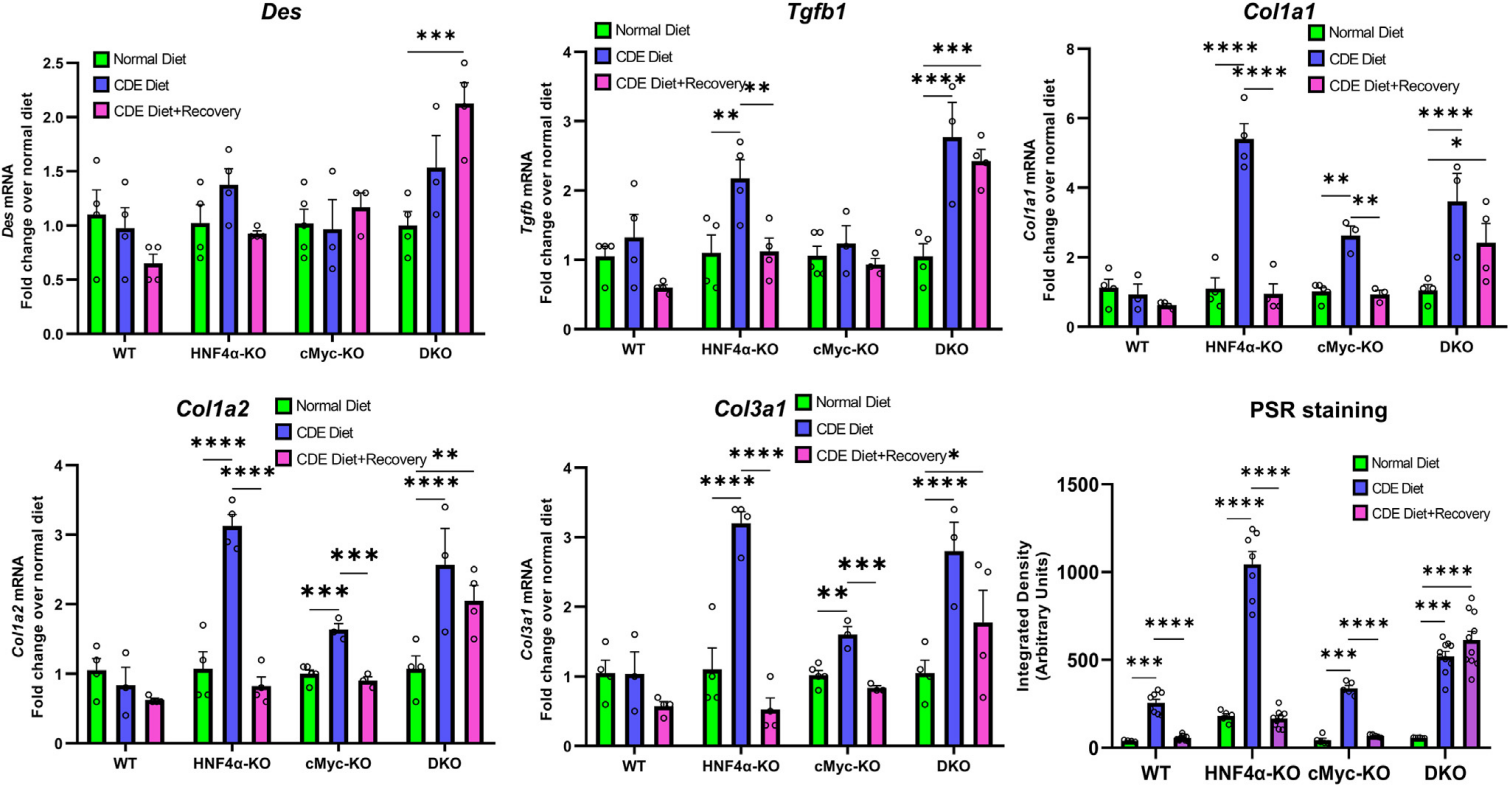
Increased fibrosis in hepatocyte nuclear factor 4 alpha (HNF4α)-knockout (KO) mice after 1 week of a choline-deficient and ethionine-supplemented (CDE) diet. Real-time PCR analysis of fibrosis markers *Des, Tgfb*, *Col1a1*, *Col1a2,* and *Col3a1* and Picrosirius red (PSR) staining quantification in wild-type (WT), HNF4α-KO, cMyc-KO, and double KO (DKO) mice after CDE diet–induced injury and regeneration. Bars represent means ± SEM. *n* = 3 to 5. ∗*P* < 0.05, ∗∗*P* < 0.01, ∗∗∗*P* < 0.001, ∗∗∗∗*P* < 0.0001.

**Figure 5:**
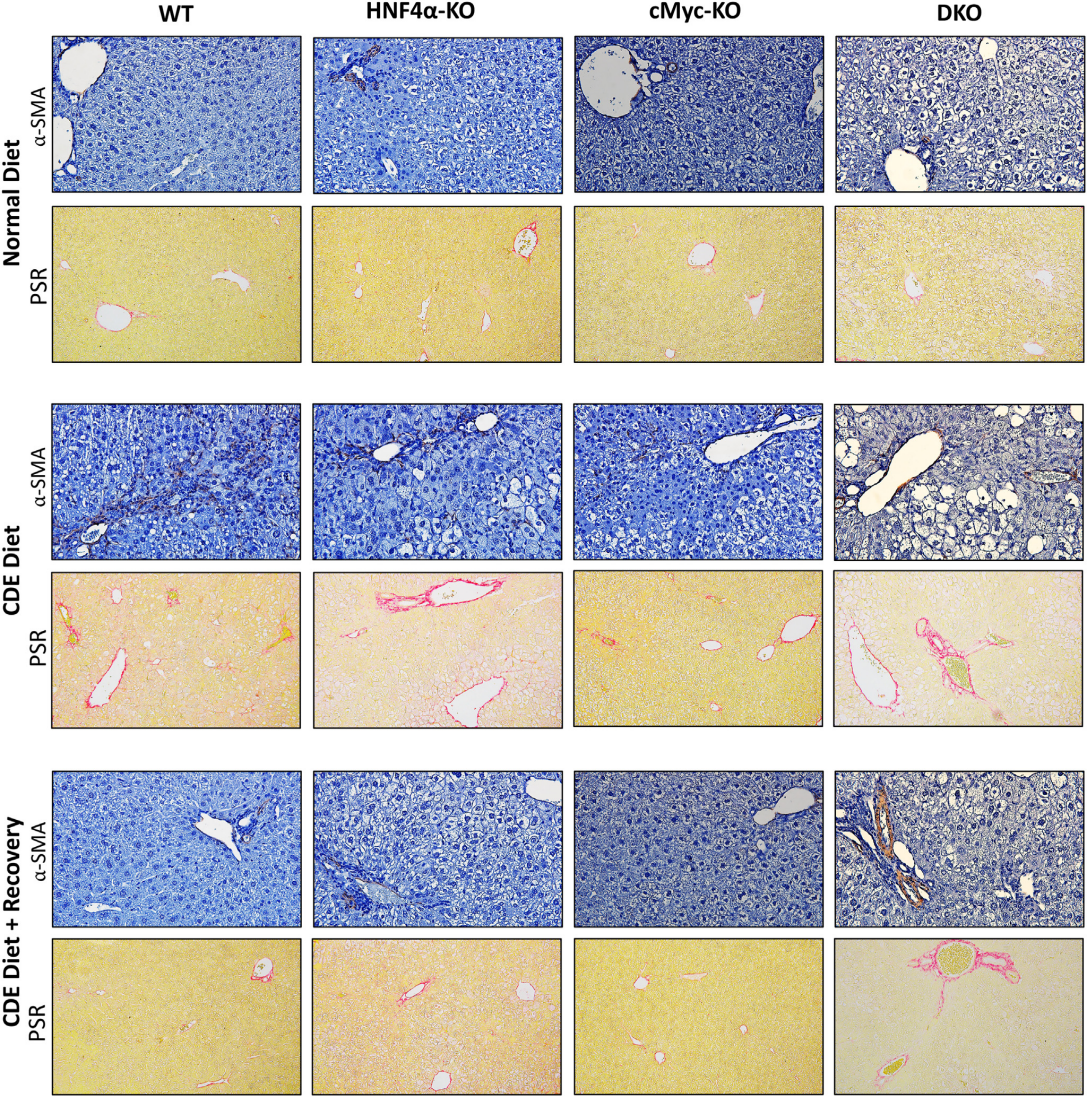
Increased Picrosirius red (PSR) staining in hepatocyte nuclear factor 4 alpha (HNF4α)-knockout (KO) mice after 1 week of a choline-deficient and ethionine-supplemented (CDE) diet. Representative photomicrographs of α-smooth muscle actin (α-SMA) immunohistochemistry and PSR staining in wild-type (WT), HNF4α-KO, cMyc-KO, and double KO (DKO) mice after CDE diet–induced injury and regeneration. Original magnification: ×400 (top rows); ×200 (bottom rows).

To investigate possible early fibrotic responses in these mice, qPCR analysis was performed of the known fibrosis markers, including *Des, Tgfb1*, *Col1a1*, *Col1a2,* and *Col3a1.* HNF4α-KO mice showed significantly higher expression of fibrosis genes during the injury phase. The model of cMyc-KO mice resulted in significant activation of collagen genes, which was lower than HNF4α-KO mice. In the DKO model, the induction of fibrosis genes was comparable to HNF4α-KO mice (Figure 4). The mRNA data were further corroborated by α-smooth muscle actin immunohistochemistry staining and Picrosirius red staining (Figure 5). During the recovery phase, increased fibrosis in HNF4α-KO and cMyc-KO mice significantly decreased. However, DKO mice exhibited consistently higher fibrosis responses, even during the recovery phase.

### Increased Proliferation in HNF4α-KO Mice after 1 Week of the CDE Diet

**Figure 6:**
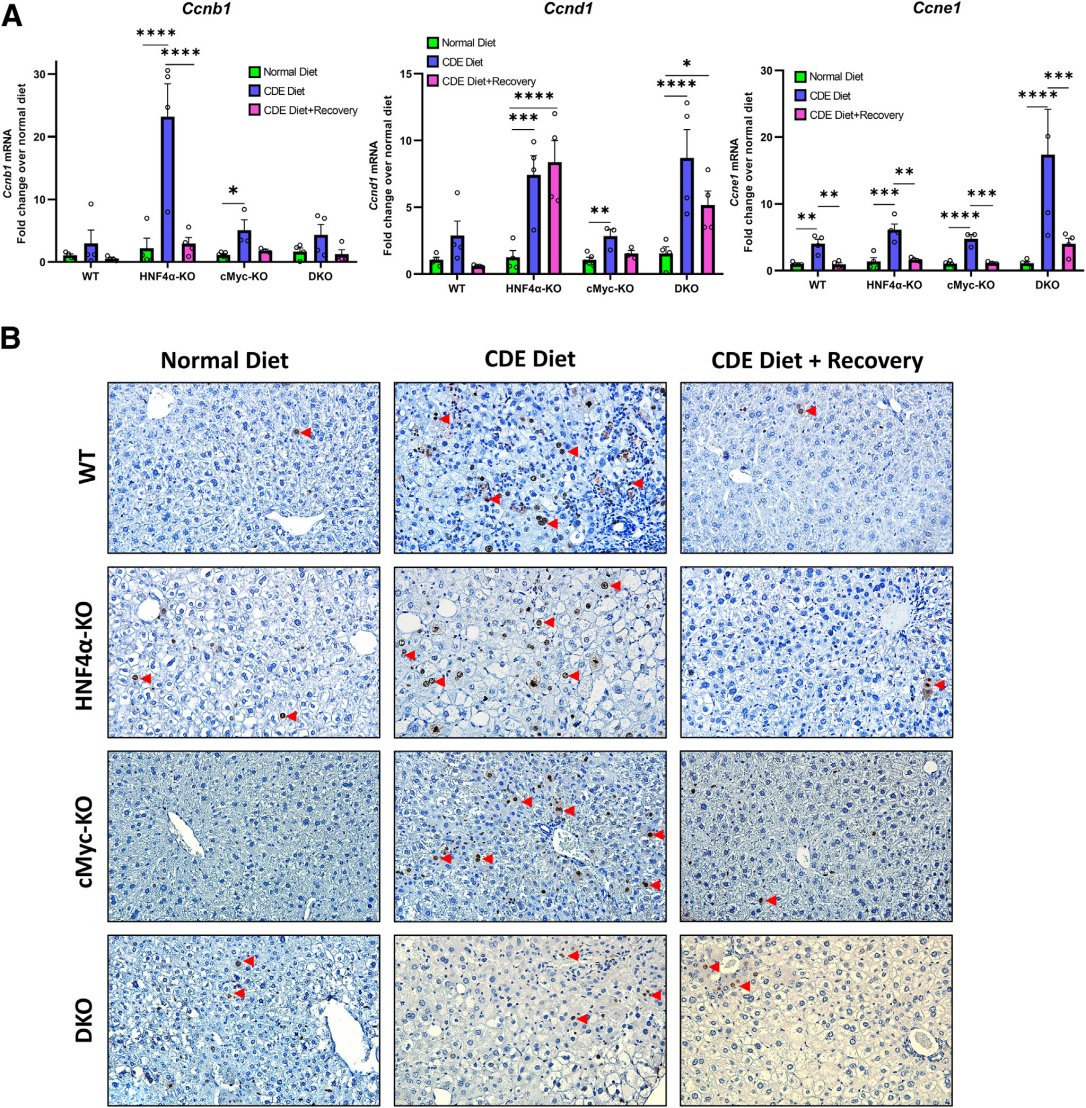
Increased proliferation in hepatocyte nuclear factor 4 alpha (HNF4α)-knockout (KO) mice after 1 week of a choline-deficient and ethionine-supplemented (CDE) diet. Real-time PCR analysis of proliferation markers *Ccnb1, Ccnd1*, and *Ccne1* (**A**) and representative photomicrographs of Ki-67 immunohistochemistry (**B**) in wild-type (WT), HNF4α-KO, cMyc-KO, and double KO (DKO) mice after CDE diet–induced injury and regeneration. **Red arrowheads** indicate Ki-67–positive cells. Bars represent means ± SEM. *n* = 3 to 5. ∗*P* < 0.05, ∗∗*P* < 0.01, ∗∗∗*P* < 0.001, ∗∗∗∗*P* < 0.0001. Original magnification: ×400.

Liver regeneration following CDE diet–induced liver injury was investigated by qPCR analysis of proliferation markers, including *Ccnb1, Ccnd1,* and *Ccne1.* WT mice exhibited minimal changes in proliferation genes, but HNF4α-KO, cMyc-KO, and DKO mice showed a significantly higher expression of cyclins during the injury phase. The increase in cyclin expression was reduced back to control levels in cMyc-KO mice during recovery. However, HNF4α-KO and DKO mice showed continued expression of cyclin genes, albeit lower than during the injury phase (Figure 6A). Ki-67 immunohistochemistry further confirmed an increased proliferation response (Figure 6B).

### Significant Increase in HPC Markers Both after 1 Week of CDE Diet–Induced Injury and after 1 Week of Recovery in DKO Mice

**Figure 7:**
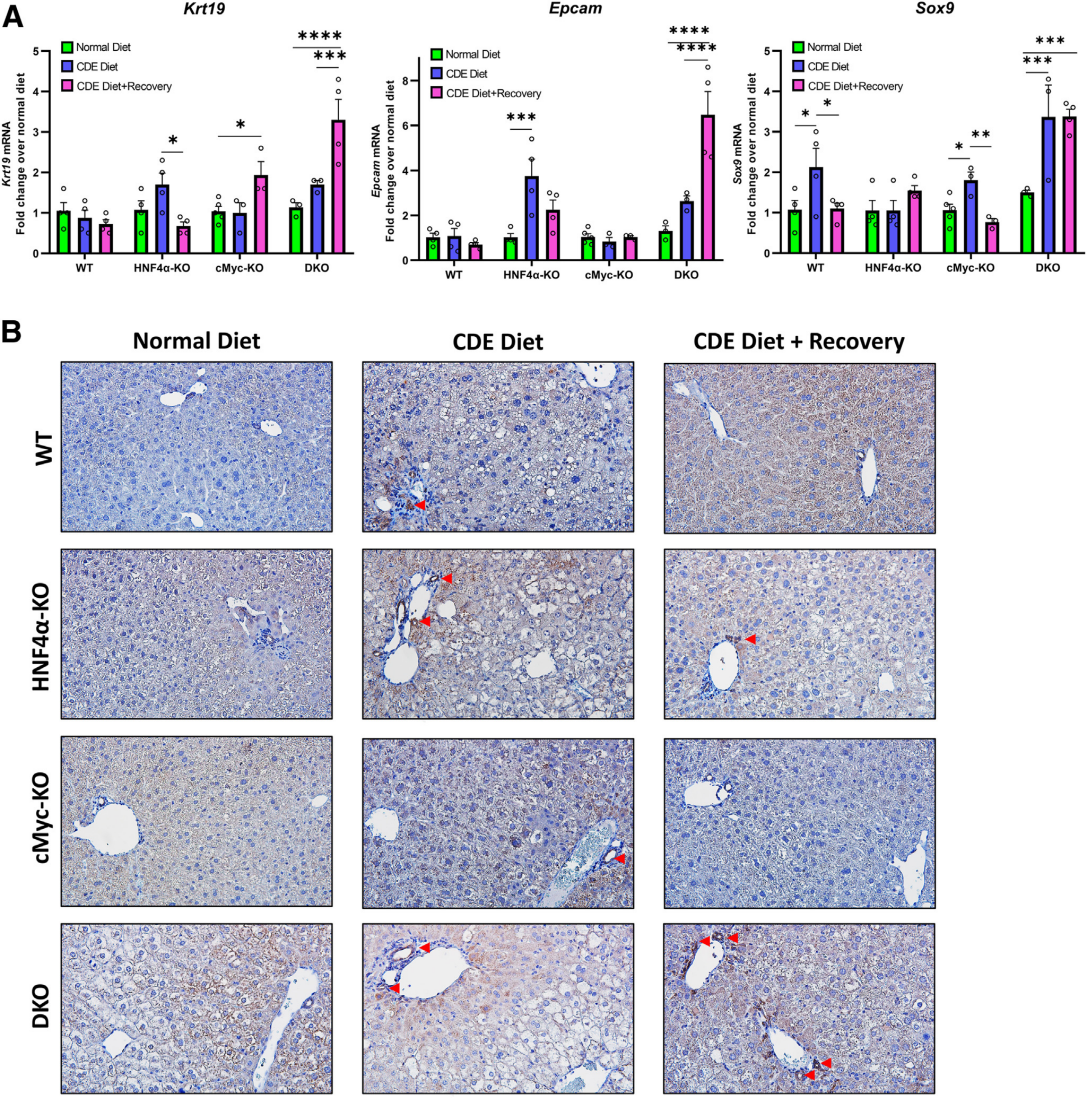
Significant increase in hepatic progenitor cell markers both after 1 week of choline-deficient and ethionine-supplemented (CDE) diet–induced injury and after 1 week of recovery in double knockout (DKO) mice. Real-time PCR analysis of *Krt19*, *Epcam*, and *Sox9* (**A**) and representative photomicrographs of Epcam immunohistochemistry (**B**) in wild-type (WT), hepatocyte nuclear factor 4 alpha (HNF4α)-knockout (KO), cMyc-KO, and DKO mice after CDE diet–induced injury and regeneration. **Red arrowheads** indicate Epcam-positive cells. Bars represent means ± SEM. *n* = 3 to 5. ∗*P* < 0.05, ∗∗*P* < 0.01, ∗∗∗*P* < 0.001, ∗∗∗∗*P* < 0.0001. Original magnification: ×400.

Finally, to investigate the HPC activation and HPC-driven proliferation response in these mice, qPCR analysis of HPC markers such as *Krt19*, *Epcam,* and *Sox9* was performed. WT mice exhibited no change in *Krt19* or *Epcam* but showed an increase in *Sox9* expression only during the injury phase. HNF4α-KO mice showed an increase in *Krt19* and *Epcam* expression only during the injury phase. cMyc-KO mice showed no change in *Epcam* and *Sox9* expression but a small increase in Krt19 only during the recovery phase. The DKO mice exhibited the most robust HPC activation response, with an increase in *Krt19* and *Epcam* expression during the recovery phase and an increase in Sox9 during both the injury and recovery phases (Figure 7A). Sox9 immunofluorescence further supported the mRNA data, exhibiting increased Sox9-positive cells in all groups following a CDE diet for 1 week (Figure 7B). The DKO mice had the highest number of Sox9-positive cells in the liver after 1 week of CDE diet. Interestingly, no Sox9-positive cells were detected during the recovery phase in DKO livers despite significantly higher *Sox9* mRNA expression.

## Discussion

The role of HNF4α, an orphan nuclear receptor expressed exclusively in hepatocytes, has been well defined in embryonic liver development, as well as for postnatal maintenance of the differentiated phenotype.^2^ HNF4α also inhibits hepatocyte proliferation and maintains a differentiated state. Loss of HNF4α results in de-differentiation and increased proliferation in the absence of liver injury.^2^ Progression of chronic liver disease is associated with loss of HNF4α activity.^11^ Recent studies explored the role of HNF4α in liver regeneration after partial hepatectomy and after acetaminophen overdose, a model of drug-induced liver injury.^3^^,^^4^ The partial hepatectomy model has relatively low injury, no inflammation, and synchronized liver regeneration, whereas the acetaminophen overdose model has necrosis and inflammation followed by unsynchronized liver regeneration. However, in both models, injury is induced acutely. Furthermore, these models do not have either the early fibrotic changes or activation of HPCs, which are the hallmark of chronic liver disease. The goal of the current study was to investigate the role of HNF4α in liver regeneration, subacute liver injury, and subsequent regeneration after CDE diet in which inflammation, fibrosis, and HPC-mediated proliferation are involved.

Two-month–old C57BL/6J mice experienced 10 times higher liver injury compared with the control mice after 1 week of a CDE diet. A significant decrease in HNF4α expression was observed at the mRNA and protein levels in these mice. However, these mice showed a significant reduction in liver injury and re-gained HNF4α expression upon recovery on a normal diet. These data suggest that maintaining HNF4α expression is necessary for recovery from CDE diet–induced liver injury. CDE diet studies in hepatocyte-specific HNF4α-KO mice supported these findings. HNF4α-KO mice experienced higher liver injury after CDE diet compared with WT mice. These mice, however, recovered and restored their histology after 1 week of recovery. Quantification of inflammation and fibrosis markers revealed that HNF4α-KO mice had higher expression of these genes during injury compared with WT mice. This finding further highlighted the significance of maintaining HNF4α expression during subacute liver injury.

The proliferation response was studied to analyze the regeneration process in these mice. Deletion of HNF4α is known to induce a spontaneous proliferation response in hepatocytes.^12^ Consistent with these findings, HNF4α-KO mice exhibited a significant induction in the expression of cyclins and Ki-67–positive cells during the injury period. These data suggest that decreased HNF4α expression during injury is associated with increased hepatocyte entry into the cell cycle, helping these mice recover via regeneration.

Deletion of HNF4α results in increased expression of cMyc at the mRNA and protein levels.^2^^,^^3^ cMyc is a proto-oncogene that is often deregulated in various cancers.^13^ cMyc is a major regulator of cell proliferation, cell cycle progression, cell growth, metabolism, and differentiation.^14^ The spontaneous proliferation and metabolic changes observed after HNF4α deletion are at least in part due to up-regulation of cMyc. We therefore investigated the role of HNF4α-cMyc interaction in liver regeneration after subacute liver injury induced by CDE diet. Deletion of both HNF4α and cMyc in DKO mice resulted in significant liver injury comparable to the HNF4α-KO mice after 1 week of CDE diet and complete recovery on a normal diet for 1 week. Increased inflammation, fibrosis, and proliferation response after CDE diet were higher than in WT mice and comparable to that in HNF4α-KO mice. DKO mice exhibited consistently higher fibrosis responses during the recovery phase. Interestingly, DKO mice showed a significant increase in HPC markers both after 1 week of CDE diet–induced injury and after 1 week of recovery. This suggests that in the case of both HNF4α and cMyc deletion, liver regeneration and recovery are driven less by hepatocyte proliferation and more by HPC activation. These data support our hypothesis that cMyc activation after HNF4α induces hepatocyte proliferation.

Deletion of cMyc in HNF4α-KO mice reduces acetaminophen-induced acute liver injury.^4^ However, in the current study of CDE diet–induced injury, in which the injury is physiologically similar to that of chronic liver diseases, deletion of cMyc in HNF4α-KO mice exacerbated HPC-driven proliferation. In humans, increased HPC activation is associated with the severity of chronic liver disease.^15^ Progressive loss of HNF4α is associated with chronic liver disease progression.^11^ Decreased HNF4α expression results in increased cMyc expression. Studies have shown that enhanced expression of cMyc promotes liver disease progression^16^, ^17^, ^18^ and increased HPC response.^19^ As a result, targeting the HNF4α-cMyc interaction could be a potential therapeutic approach for treating chronic liver disease.

In summary, deletion of HNF4α increased subacute liver injury induced by CDE diet, and deletion of cMyc protects against the injury. Deletion of HNF4α resulted in increased inflammation, fibrosis, proliferation, and HPC activation, all of which (except inflammation) are reduced after cMyc deletion. Deletion of both HNF4α and cMyc resulted in a phenotype similar to that of HNF4α deletion after CDE diet; however, it involved HPC-driven regeneration response to recover from injury. Taken together, these data show that HNF4α protects against inflammatory and fibrotic changes after CDE diet–induced injury, which is driven by cMyc.

## Disclosure Statement

None declared.
